# Facile synthesis of magnesium-based metal-organic framework with tailored nanostructure for effective volatile organic compounds adsorption

**DOI:** 10.1098/rsos.211544

**Published:** 2022-03-30

**Authors:** Zichu Hu, Hui Liu, Ya Zuo, Yufei Ji, Shujing Li, Wanqi Zhang, Zhechen Liu, Zhangjing Chen, Xiaotao Zhang, Ximing Wang

**Affiliations:** ^1^ College of Science, Inner Mongolia Agriculture University, Hohhot 010018, People's Republic of China; ^2^ College of Material Science and Art Design, Inner Mongolia Agriculture University, Hohhot 010018, People's Republic of China; ^3^ Department of Sustainable Biomaterials, Virginia Polytechnic Institute and State University, Blacksburg, VA 24060, USA; ^4^ Inner Mongolia Key Laboratory of Sandy Shrubs Fibrosis and Energy Development and Utilization, Hohhot, 010018, People's Republic of China

**Keywords:** magnesium-based metal-organic framework, adsorption, wood drying, VOCs

## Abstract

A novel Mg(II) metal-organic framework (Mg-MOF) was synthesized based on the ligand of 2,2'-bipyridine-4,4'-dicarboxylic acid. Single-crystal X-ray structural analysis confirmed that three-dimensional-nanostructure Mg-MOFs formed a monoclinic system with a channel size of 15.733 Å × 23.736 Å. N_2_ adsorption isotherm, Fourier transform infrared spectroscopy, thermogravimetric analysis and high-resolution transmission electron microscopy were performed to characterize the thermal stability and purity of the Mg-MOFs. The adsorption studies on four typical volatile organic compounds (VOCs) emitted during wood drying showed that Mg-MOFs have noteworthy adsorption capacities, especially for benzene and β-pinene with adsorptions of 182.26 mg g^−1^ and 144.42 mg g^−1^, respectively. In addition, the adsorption of Mg-MOFs mainly occurred via natural adsorption, specifically, multi-layer physical adsorption, accompanied by chemical forces, which occurred in the pores where the VOCs molecules combined with active sites. As an adsorbent, Mg-MOFs exhibit versatile behaviour for toxic gas accumulation.

## 1. Introduction

With rapid industrialization and urbanization, atmospheric pollution has always remained one of the most serious environmental issues and has also attracted wide attention [1–3]. Volatile organic compounds (VOCs) are defined as a class of organic compounds with a saturated vapour pressure of 0.01 KPa or more at 293.15 K, or having a corresponding volatility under the particular conditions of use [4]. VOCs cause adverse impacts on human health because of their genotoxic and carcinogenic effects [5–7]. VOCs are also causes of the formation of fine particulate matter [8,9], the destruction of the ozone layer and photochemical smog [10]; furthermore, they aggravate the greenhouse effect [11]. The main sources of VOCs are from various industrial processes [12,13], especially wood drying, from which many VOCs are released, typically including terpenes, aldehydes, haloalkanes, aromatic hydrocarbons and alcohols [14,15]. Therefore, the elimination of VOCs in the wood-drying process is a major concern for the development of effective treatment technologies [16–19].

Traditional methods, such as condensation [20], catalytic oxidation [21,22], photocatalysis [23], adsorption and absorption, have been applied extensively to treat and purify VOCs [24–26]. Among these, adsorption is extremely convenient owing to its characteristic advantages, such as ease of fabrication, effectiveness, low cost, stability and high efficiency [27]. Hence, the critical factor in the adsorption method is choosing a suitable adsorbent for high-performance VOC capturing [28,29].

Metal-organic frameworks (MOFs) have shown significant potential in separation, adsorption, sensing and catalytic applications owing to their ordered crystalline nanostructure, high selectivity, large adsorption capacity, low-temperature desorption and stable crystal structures after regeneration [30–33]. MOFs are exploited for their structural features, including the pore polarity, porosity, coordination model and ligands with special functional groups [34], which can be designed to form specific interactions between the MOF's surface and the targeted pollutant molecules [35–38]. In MOFs, metal ions and organic-unit linkers both enable its structural transformations to occur upon the adsorption of VOC molecules throughout the rearrangement of the flexible organic linkers, resulting in a well-established adsorption mechanism [39]. Thus, rational designs for high-efficiency MOFs to adsorb harmful VOCs arising from the wood-drying process are attracting interest in light of the crystal nanostructure's stability and appropriate pore size [40–43]. Among them, Mg-MOF has many applications. Reda Salama *et al*. [44] prepared Mg-MOF for catalytic applications in the synthesis of coumarin and dihydropyrimidinone derivatives. Mallick *et al*. [45] prepared Mg-MOF for selective adsorption. CO_2_ and H_2_ have a good effect. The Mg-MOF-74 prepared by Qasem *et al*. K [46] has good adsorption capacity and cycle stability for CO_2_.

**Scheme 1.**
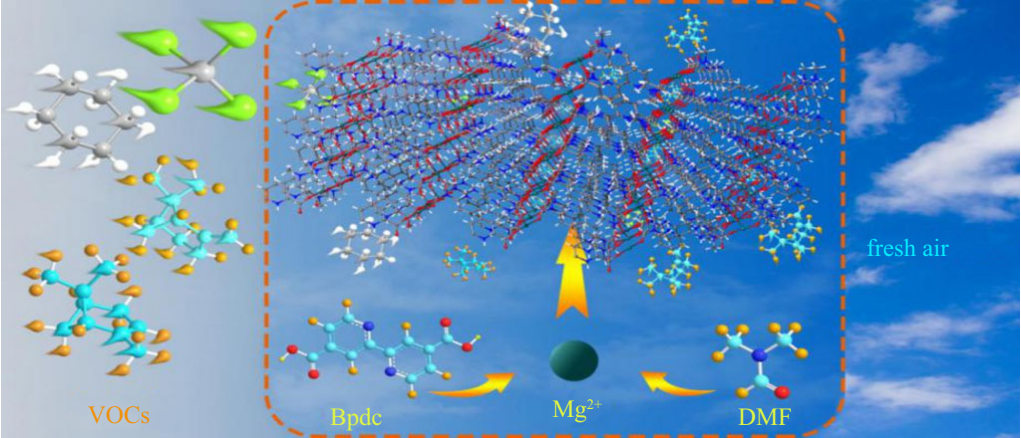
Structures of Mg-MOFs and their VOCs adsorption efficiency.

This work attempts to incorporate a novel three-dimensional magnesium MOF (Mg-MOF), obtained from 2,2′-bipyridine-4,4′dicarboxylic acid (Bpdc) and Mg(II) in N,N-dimethylformamide (DMF) through a solvothermal approach, as a new example of Bpdc- and alkaline-earth-metal-based MOFs. Comprehensive characterization results showed that the particular arrangement of the organic-ligand Bpdc and DMF within Mg-MOFs, combined with strong O-to-Mg coordination, contribute to the formation of its pore structure, comprising rhombic channels with [Mg_2_(COO)_2_] as the vertices and Bpdc as the edges with diagonal distances of 15.733 Å × 23.736 Å. To investigate the adsorption properties of Mg-MOFs, four harmful gases, namely, benzene, β-pinene, α-pinene and tetrachloromethane, with high-release values during wood drying were chosen as typical VOC adsorbates. Static adsorption tests were carried out to reveal the effects of the relative pressure and time on the adsorption efficiency of these VOCs. The findings demonstrated that Mg-MOFs show promising adsorption capacity of the VOCs studied; in particular, the adsorption capacities of benzene and β-pinene are remarkable. Furthermore, the possible adsorption mechanisms are also discussed, which may involve effective π−π stacking interactions, hydrogen bonding, instantaneous dipole forces, dispersion forces, London forces and polar effects. The three-dimensional MOF structures of the Mg-MOFs and their efficiency in adsorbing VOCs from wood drying are shown in scheme 1.

## 2. Experimental

### 2.1. Material and methods

All the chemicals and reagents used were of analytical grade. Magnesium (II) chloride hexahydrate (MgCl_2_·6H_2_O) was purchased from Tianjin Fengchuan Chemicals Co. Ltd. (Tianjin, China), Bpdc was purchased from Aladdin (Aladdin Industrial Cooperation, Shanghai, China) and DMF was obtained from Tianjin Chemical Reagent Co., Ltd. (Tianjin, China).

#### 2.1.1. Synthesis of magnesium-metal-organic frameworks

Mg-MOFs with the formula [Mg(Bpdc)(DMF)_2_]n were prepared through the solvothermal method. Specially, MgCl_2_·6H_2_O was placed in a muffle furnace (KSL-1100X, Hefei, China) and dried at 350°C for 6 h to remove water from the crystal. MgCl_2_ (0.0595 g, 0.625 mmol) was dispersed in DMF (5 ml) in a beaker and Bpdc (0.25 mmol, 0.0610 g) was dispersed in the solution, followed by ultrasonication for 30 min at 25°C. Next, the mixture was transferred to a 25 ml Teflon-lined autoclave in a steel Parr vessel. The Parr vessel was sealed and dried in a vacuum oven (DZ-1BC II, Tianjin, China) under autogenous pressure to 120°C at 30°C h^−1^ for 48 h, then cooled to 30°C at 2.0–2.5°C h^−1^ to yield colourless crystals. The precipitation was centrifuged (6000 rpm) (H2050R, Hunan, China) and washed five times with DMF, and the colourless transparent crystals were obtained. Afterwards, the product was activated in a vacuum oven (DZ-1BC II, Tianjin, China) at 60°C for 24 h prior to further analysis. A schematic reaction scheme for the Mg-MOFs synthesis is shown in the electronic supplementary material, figure S1.

#### 2.1.2. Characterization

Crystallographic data of Mg-MOFs ([Mg(Bpdc)(DMF)_2_]_n_) were collected on a Bruker SMART CCD system (Bruker, German) equipped with monochromated Mo-Ka radiation (*λ* = 0.71073 Å) using the *ω*−*φ* scan technique. Data integration and empirical absorption corrections were carried out by SAINT programmes, and the structures were solved by direct methods (SHELXS 97). The non-hydrogen atoms were refined anisotropically on F2 via full-matrix least-squares techniques (SHELXL 97) [47]. A PANalytical empyrean sharp shadow system X-ray diffractometer was used to get the X-ray diffraction (XRD) patterns of the samples over the 2*θ* range of 5°–80° and Cu Ka radiation (*λ* = 1.540598 Å) was used during the testing procedure. All the hydrogen atoms, except for those of the uncoordinated water molecules, in these coordination polymers were generated geometrically and refined isotropically using the riding model. The detailed crystal parameters, data collection and refinements for Mg-MOFs are summarized in table 1. The selected bond lengths and angles of Mg-MOFs are presented in table 2.

**Table 1.** Crystallographic data for Mg-MOFs.

formula	C_18_H_22_N_4_O_6_Mg
Fw(g mol^−1^)	414.704
T (K)	296
crystal system	monoclinic
space group	C2/c
a (Å)	23.736(4)
b (Å)	8.9565(16)
c (Å)	9.2378(18)
*α* (°)	90
*β* (°)	93.349(4)
*γ* (°)	90
D_calcd_/g cm^−3^	1.405
V (Å^3^)	1960.5(6)
Z	4
*μ* (mm^−1^)	0.134
F (000)	872.0
reflections collected	6573
*R*_1_, wR_2_ [*F*^2^ > 2*σ*]	*R*_1_ = 0.0891, wR_2_ = 0.2375
*R*_1_, wR_2_ (all data)	*R*_1_ = 0.1258, wR_2_ = 0.2705
GOF on *F*^2^	1.024
largest difference peak and hole (e Å^−3^)	1.31, −0.76

**Table 2.** Selected bond lengths and bond angles for Mg-MOFs.

bond lengths (Å)					
Mg1-O1	2.040(3)	Mg1-O2	2.079(3)	Mg1-O3	2.120(5)
bond angles (°)					
O1-Mg1-O1A	90.7(2)	O1-Mg1-O2	94.72(13)	O1A-Mg1-O2B	93.57(14)
O2B-Mg1-O2C	168.2(3)	O1-Mg1-O3	175.98(18)	O1-Mg1-O3A	87.00(17)
O2B-Mg1-O3A	89.88(18)	O3-Mg1-O2B	82.16(17)	O3-Mg1-O3A	95.5(3)

The specific surface area and porous system of the obtained adsorbents were characterized based on the N_2_ adsorption/desorption isotherm (ASAP 2020, Micrometrics, Norcross, GA, USA). The Fourier transform infrared (FTIR) spectrum (KBr pellets as substrate) was characterized on an FTIR spectrometer (Thermo Nicolet Nexus; Thermo Fisher Scientific, Waltham, MA, USA). The thermographic analyses (TGA) of Mg-MOFs were performed on a TGA analyser (Q600, Ta Instruments, Inc., USA). The temperature was increased from 25°C to 800°C at a heating rate of 5°C min^−1^ under a 20 ml min^−1^ airflow. High-resolution transmission electron microscopy (HRTEM) images of the samples were acquired using an HRTEM (FEI Talos F200X, Thermo Fisher Scientific, USA) at 300 kV, and the samples were drop-dried on carbon-coated microgrids. Energy-dispersive X-ray spectroscopy (EDX) analysis was performed (JEM-2010, Tokyo, Japan). Gaseous VOCs adsorption and desorption experiments were performed on an intelligent gravimetric analyser (3H-2000PW, BeiShiDE Instrument Technology Beijing Co. Ltd., China) and RUBOTHERM magnetic suspension balance (Bochum, Germany), respectively.

### 2.2. Volatile organic compounds adsorption/desorption experiments

Static VOCs adsorption/desorption tests were carried out via 3H-2000PW manufactured by BeiShiDE, China (electronic supplementary material, figure S2). A 100 mg of Mg-MOFs were placed in the sample cell and heated to 300°C under high vacuum to remove impurities and moisture. High-release VOCs vapour (benzene, β-pinene, α-pinene and tetrachloromethane) was treated with vacuum degassing, was released into the test chamber and the pressure in the test chamber and change in Mg-MOF mass were recorded continuously. The VOCs concentration in the outlet gas stream as a function of adsorption time, was plotted as a breakthrough curve, assuming 10% of the feed concentration as the breakthrough point. The equilibrium adsorption capacity (*q*) of the Mg-MOFs was calculated from the breakthrough curve according to equation (2.1) [48]:

2.1 $$q=\frac{F}{m}\left(C_{0}t_{f}-\int_{0}^{tf}C_{t}\;dt\right),$$

where *F* (L min^−1^) is the VOCs gas-flow rate, *m* (g) is the mas of Mg-MOFs loaded in the adsorption cell, *C*_0_ (mg l^−1^) is the feed concentration of the VOCs, *t*_f_ (min) is the adsorption time and *q* (mg g^−1^) is the adsorption capacity.

The desorption tests were carried out in the same set-up with Mg-MOFs saturated by VOCs at 25°C; the adsorbents were then cooled down and manipulated using a vacuum pump. The outlet concentration of desorbed VOCs was continuously monitored by the restore atmospheric pressure valve and the desorption patterns were obtained by an online gas chromatograph.

## 3. Results and discussion

### 3.1. Crystal structure of magnesium-metal-organic frameworks

**Figure 1.**
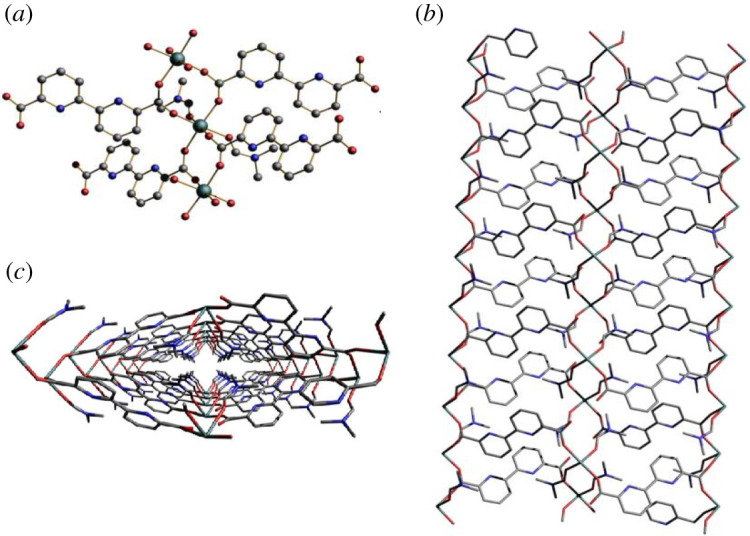
(*a*) Coordination environment for Mg-MOFs, the hydrogen atoms are omitted for clarity; three-dimensional MOF structures of Mg-MOFs along the (*b*) a axis and (*c*) c axis. H atoms are omitted for clarity. Colour code: dark green (Mg), red (O), blue (N), grey (C).

X-ray crystallography reveals that the Mg-MOFs ([Mg(Bpdc)(DMF)_2_]_n_) crystallize in a monoclinic system, with space group C2/c. As shown in figure 1*a*, the central Mg(II) is octahedrally coordinated by four oxygen atoms through monodentate coordinating mode from four different carboxylate groups with different Bpdc ligand and two adjacent oxygen atoms from two DMF molecules. The Bpdc ligand, as a linker with each of the carboxylate groups, bridges two Mg(II) ions to form a three-dimensional framework with one-dimensional channels (figure 1*b*). Channels can be seen clearly along the c-axis (figure 1*c*). The size of the channel is 15.733 Å × 23.736 Å, and its crystallographic parameters are listed in tables 1 and 2.

**Figure 2.**
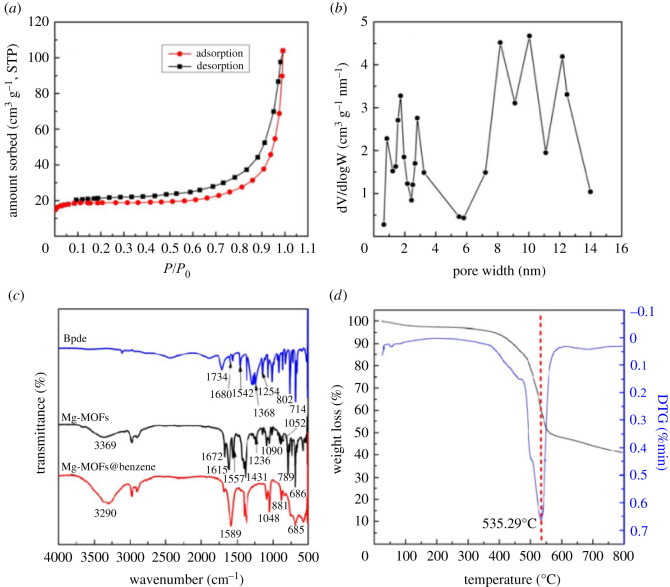
(*a*) N_2_ adsorption/desorption isotherms at 77 K; (*b*) pore-size distributions of Mg-MOFs; (*c*) FTIR spectra of Bpdc, Mg-MOFs and Mg-MOFs@benzene; and (*d*) TGA curves of Mg-MOFs.

The porosity of synthesized Mg-MOFs was recorded using N_2_ adsorption/desorption isotherms at 77 K (figure 2*a*,*b*). As shown in figure 2*a*, the shape of the Mg-MOF isotherm was a combination of type-I (pore size less than 2 nm) and type-IV (2 nm < pore size less than 50 nm) isotherms with an apparent hysteresis loop, as defined by IUPAC [49,50], which suggests the coexistence of micropores and mesopores. In general, microporous and mesoporous materials are able to capture low concentrations of small VOC molecules, and pore-filling occurs frequently at low partial pressure. Furthermore, to precisely evaluate the pore characteristics of Mg-MOFs, pore-size determination was carried out via nonlocal density functional theory methods [51]. The pore-size distributions are presented in figure 2*b*. It can be seen that there are pores at 0.7–4.1 nm and 8.0–13.2 nm, indicating that the Mg-MOFs might be mesoporous material. This pore-size distribution pattern further demonstrated the presence of a nanoscale porous structure in the single crystal. The surface area and pore volume of the Mg-MOFs are listed in the electronic supplementary material, table S1, and the Langmuir surface areas (*S*_Langmuir_) and Brunauer–Emmett–Teller (BET) surface areas (*S*_BET_) reached 829.47 m^2^ g^−1^ and 625.45 m^2^ g^−1^, respectively. Furthermore, the Mg-MOF samples showed much higher micropore (0.08 cm^3^ g^−1^) and mesopore (0.14 cm^3^ g^−1^) volumes with an average pore diameter of 1.99 nm. These results confirmed that the as-synthesized Mg-MOFs are porous, which helps them adsorb industrial wood-drying VOCs.

FTIR analysis was used to examine the characteristic chemical structures of Bpdc and Mg-MOFs and the adsorption of benzene (Mg-MOFs@benzene) samples. These data are recorded in figure 2*c*. Owing to its good effect on adsorption capacity in the experiments, we used benzene as an example to investigate the adsorption mechanism. As shown in figure 2*c*, the broad peak at 3369 cm^−1^ is the characteristic absorption peak of the intramolecular-associated hydrogen bond. After benzene was adsorbed, the peak blue shifted to 3290 cm^−1^, which may have been owing to the rapid uptake of atmospheric water during the measurement in air. Obviously, the peak at 1734 cm^−1^, attributed to the -COO stretching vibration of Bpdc, disappeared in Mg-MOFs, which suggests that a chelating coordination model of Mg-O was formed. In Mg-MOFs, 1672 cm^−1^ and 1615 cm^−1^ were the stretching vibration absorption peaks of pyridine ring C=C and C=N, and after adsorption of benzene, the peaks were apparently weakened. The 1557 cm^−1^ is attributed to the C=O asymmetric stretching vibration absorption peak, which is enhanced and moved to the vicinity of 1589 cm^−1^ after adsorption of benzene, confirming that there were hydrogen bonds between the lone electron pair of the O atom and benzene ring in the structure of the adsorbent. At 1236 cm^−1^ (in Mg-MOFs), there is an absorption peak of O-H deformation vibration peak, which almost disappears after adsorbing benzene (in Mg-MOFs@Benzene). The characteristic peaks at 1090 cm^−1^ and 1052 cm^−1^ assigned to C-C vibration absorptions of bipyridine rings were strengthened around 1048 cm^−1^ after benzene adsorption, indicating the π–π stacking and instantaneous dipole forces between the pyridine and benzene ring (in Mg-MOFs@Benzene), which results in the interactions of aromatic ring structures being sharply enhanced [52]. The peak at 789 cm^−1^ and 686 cm^−1^ (in Mg-MOFs) ascribed to C-H out-of-plane and in-plane bending vibration absorption in an aromatic ring, were shifted to 881 cm^−1^ and 685 cm^−1^ after the adsorption of benzene (in Mg-MOFs@Benzene). The information observed from the FTIR spectra indicated that the O atoms in Bpdc's -COO formed the new Mg-O bonds with Mg(II) in Mg-MOFs; furthermore, various chemical interactions, such as the π–π stacking effect, intermolecular dispersion force, dipole force between pyridine and benzene and instantaneous interaction between π electrons of benzene and Mg(II) were observed. Consequently, the absorption intensity and positions of the absorption vibration peaks of the corresponding active functional groups notably changed. The results revealed that the adsorption process of the Mg-MOFs for benzene entails both chemical-bonding effects and physical porous adsorption.

TGA spectroscopy is a powerful method that has been used to measure gases evolved during the thermal treatment of various compounds [53], including the thermal stability of the Mg-MOFs, as shown in figure 2*d*. There is less than 2.6% weight loss observed at 160°C, indicating that the treatment moved the residual solvent and minimally adsorbed moisture. The small weight loss between 160°C and 345°C is consistent with the loss of surface adsorbed DMF (approx. 1.3%). The weight loss that occurred between 398°C and 573°C is attributed to the decomposition of the organic framework (approx. 47.1%). It can be seen that the mass-loss rate reached its maximum at 535.29°C, indicating that the skeleton structure of Mg-MOFs completely collapses and decomposes at this temperature. Finally, at high temperatures, it stabilized owing to the metallic oxides generated. Therefore, the obtained Mg-MOF adsorbent can be used stably at higher temperatures and exhibits good thermal stability.

**Figure 3.**
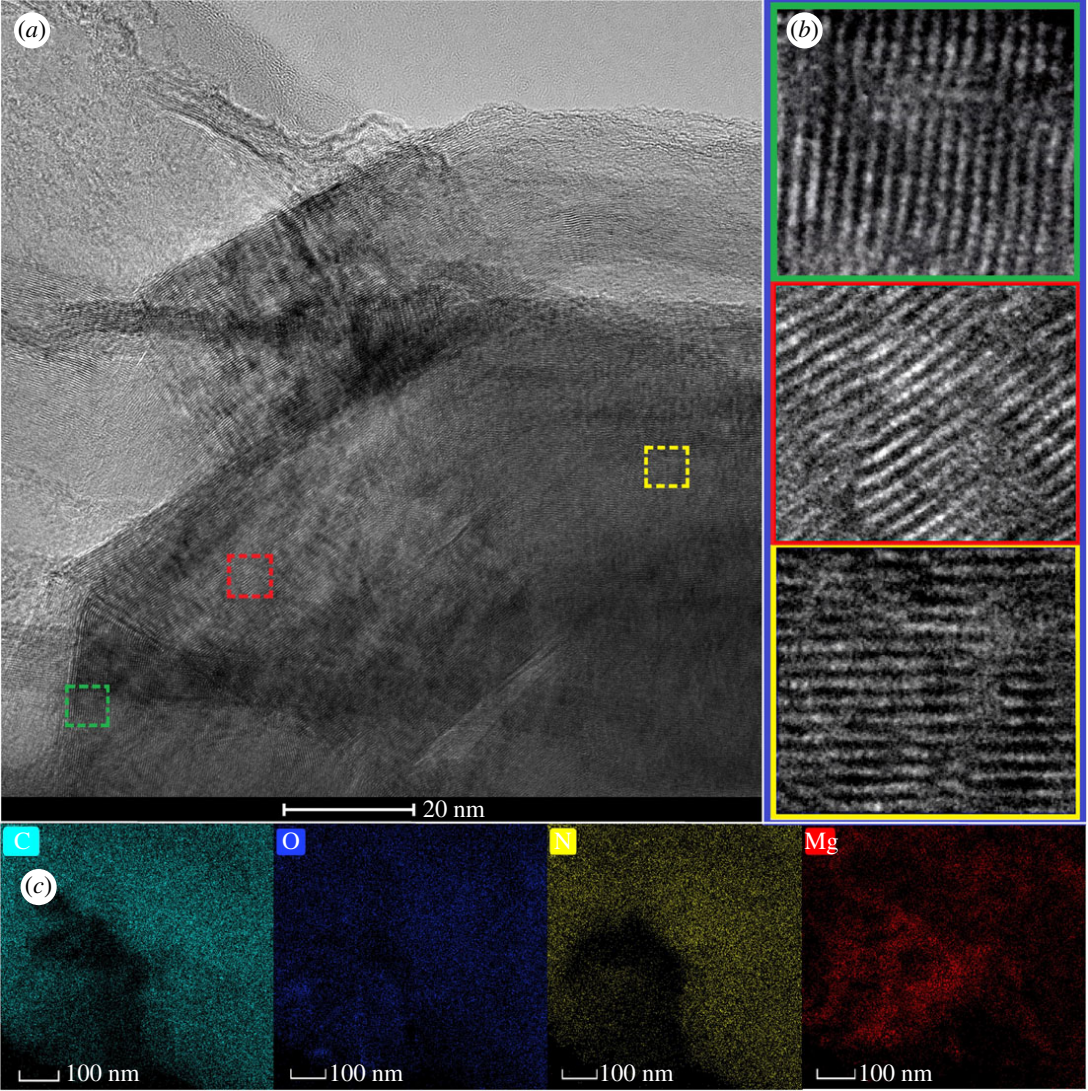
Characterizations of Mg-MOFs single crystal by HRTEM and EDX mapping. (*a*) Visualization of lattice fringes of Mg-MOFs under HRTEM. (*b*) Zoomed-in HRTEM images of three distinct regions of Mg-MOFs single crystals shown in (*a*) (green, red and yellow, respectively). (*c*) EDX-mappings of Mg-MOFs.

The HRTEM images in figure 3 show the nanoscale three-dimensional Mg-MOF single-crystal material, which was synthesized by a temperature-programmed method in this work. From figure 3*a*, it can be seen that the lattice fringes of the whole crystal are continuous and there is no obvious crystal boundary, indicating that Mg-MOFs is an integrated single crystal. Three regions in the Mg-MOF single crystal were randomly selected for FTIR observation, from which the diffraction patterns of the three regions were found to be nearly identical, which further demonstrates that all parts of the single crystal are homologous (figure 3*b*). HRTEM images revealed that the classical stacking modes and the interlaminar arrangement of zigzag were developed in Mg-MOFs single crystal [54]. In addition, many micropores and nanopore channels can be found in the structure (figure 3*a*,*b*), which can dramatically increase the specific surface areas and the porosity of Mg-MOFs, in favour of improving the adsorption of VOCs from wood drying (benzene, β-pinene, α-pinene, tetrachloromethane, etc). Furthermore, EDX mapping of the Mg-MOFs was used to verify the presence of Mg(II) ions in figure 3*c*. Based on figure 3*c*, the overlap between Mg and O is obvious, further suggesting the successful formation of Mg-O chemical bonds in Mg-MOFs. To confirm the chemical composition of Mg-MOFs, EDX analysis of the Mg-MOFs was performed (electronic supplementary material, table S2). The theoretical calculated values on the empirical formulae of Mg-MOFs showed the presence of C (52.17%), O (23.19%), N (13.53%) and Mg (5.79%), and the measured values by EDX analysis at % of Mg-MOFs were 54.34% C, 22.47% O, 12.54% N and 6.49% Mg. In short, the theoretically calculated and experimental results by EDX analyser are consistent with metal-to-linker molar ratios of 1 (Mg): 1 (Bpdc): 2 (DMF), which showed that each Mg(II) ion was coordinated with six oxygen atoms to form a three-dimensional octahedral configuration. XRD patterns of simulated Mg-MOFs and as-prepared Mg-MOFs used as materials are shown in the electronic supplementary material, figure S3. Visibly, the positions of the diffraction peaks of Mg-MOF and as-prepared Mg-MOFs used as the materials correspond well with the simulated pattern and no impurity peaks are observed, which means the as-prepared Mg-MOFs used as the materials maintained the same crystal structures of Mg-MOFs. Compared with the simulated pattern of Mg-MOFs, the broad peak width of the material is owing to the presence of VOCs molecules in the framework of Mg-MOF crystals. No other diffraction peaks are observed, indicating that no other crystals are present in the material.

### 3.2. Adsorption performance for wood-drying volatile organic compounds

**Figure 4.**
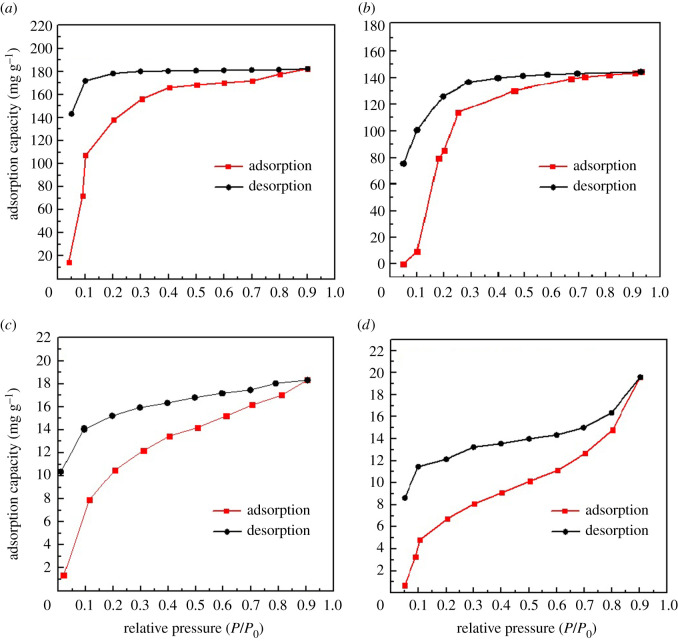
VOCs adsorption isotherms of Mg-MOFs at 298 K: (*a*) benzene; (*b*) β-pinene; (*c*) α-pinene and (*d*) tetrachloromethane.

To further elucidate the adsorption mechanism, various VOCs static adsorption/desorption isotherms were performed at 298 K (figure 4). In the full adsorption process shown in figure 4, it can be found that rising trends of benzene, β-pinene, α-pinene and tetrachloromethane were very similar: increasing rapidly in the initial stages, then gradually ascending with prolonged adsorption time. Further, the maximum adsorption capacity exhibited around *P*/*P*_0_ of 0.9 until equilibrium was reached. From figure 4*a* (red line), at low relative pressure (0 < *P*/*P*_0_ < 0.4), the isotherm showed a steep rise in uptake corresponding to the introduction of benzene molecules (dimensions 0.75 nm × 0.67 nm × 0.32 nm, molecular diameter 0.68 nm) to the surface of Mg-MOFs. The physical narrow micropore adsorption (with a 15.733 Å × 23.736 Å of Mg-MOFs, table 1) and single-layer adsorption processes were dominant on the surface, thus increasing the adsorption rate. Afterwards, the nanopores and functional sites on the surface of the Mg-MOFs were occupied (0.4 < *P*/*P*_0_ < 0.9) and the multi-layer adsorption, capillary condensation, π–π electron donor–acceptor interaction and hydrogen bonding were engendered. Thus, the adsorption amount gradually increased followed by a plateau, until the maximum adsorption capacity was 182.26 mg g^−1^ under *P*/*P*_0_ = 0.9. The results revealed that the amount of benzene adsorbed by the Mg-MOFs was positively correlated with *P*/*P*_0_. Furthermore, during the desorption phase (figure 4*a*, black line), as *P*/*P*_0_ decreased, partial benzene molecules were found to separate from Mg-MOFs and the desorption capacity displayed a slow and stable decreasing trend. As shown in figure 4*a*, the curves of the adsorption/desorption isotherm of Mg-MOFs for benzene molecules obviously could not be overlapped. Moreover, when *P*/*P*_0_ was close to 0.1, a large number of benzene molecules still could not be separated from the micropores and the surface of the Mg-MOFs, indicating that the adsorption of benzene comprises not only physical micropore adsorption, but also different chemical interactions, such as the coordination effects of π electrons in benzene and the empty electron orbits (3*s*_0_ and 3*p*_0_) in Mg (II), π–π stacking interactions between benzene and pyridine heterocycles, hydrogen bonding, instantaneous dipole forces, dispersion forces and London forces, which led to an irreversible adsorption process [55].

Comparatively, the adsorption/desorption processes of β-pinene and α-pinene on Mg-MOF are also demonstrated in figure 4*b*,*c*. It is clear that the adsorption capacity trends of both β-pinene and α-pinene first increased sharply with increased relative pressure, then remained at a slow ascent until the adsorption reaches saturation, at which time the adsorption amount is the maximum around *P*/*P*_0_ = 0.9. These results revealed that the adsorption process can be divided into two stages as the relative pressure increases: (i) the physical adsorption in the narrow micropores and the single-layer functional sites chemical adsorption between VOCs and Mg-MOFs, and (ii) the multi-layer chemical-bonding adsorption, capillary condensation and π–π electron donor–acceptor interactions, which were similar to the process of adsorption of benzene molecules. Interestingly, the adsorption amount of β-pinene 144.42 mg g^−1^ was significantly higher than that of α-pinene, 18.32 mg g^−1^. This may be attributed to the different position of C=C in the structure, which was the primary adsorption interaction between pinene and pyridine, because C=C was exposed to the β-pinene surface and was prone to chemical reactions, whereas C=C in α-pinene was inside the hexatomic ring and thus difficult to react chemically, although the molecular diameters were similar (0.72 nm of β-pinene versus 0.75 nm of α-pinene) [51]. In other words, steric hindrance greatly influences the adsorption effect. Meanwhile, the desorption trends both gradually declined and was parallel to that of benzene (figure 4*a*), and the adsorption process was also irreversible. These results imply that the adsorption process of pinene on Mg-MOFs is mainly controlled by chemical adsorption interactions. The saturated loading of Mg-MOF-74 prepared by Liu *et al*. [56] reached 8.2 mmol g^−1^ for benzene. The adsorption capacity of Mg-MOF-74 prepared by Yao *et al*. [57] for tetrachloromethane reached 30.3 cm^3^ g^−1^.

To explore the adsorption mechanism, especially π–π stacking interactions between VOCs and Mg-MOFs, we have chosen tetrachloromethane, another VOC with a higher percentage content [42] from the wood-drying process in this work (figure 4*d*). It is be learned that tetrachloromethane has an approximate molecule diameter 0.60 nm, while there are no C=C bonds in its structure. It indicated that the variation trends of the adsorption/desorption process were similar to that of benzene, β-pinene and α-pinene. However, the adsorption capacity was very low (approx. 19.60 mg g^−1^), which may be owing to the lack of strong π–π stacking interactions between Mg-MOFs and tetrachloromethane. In brief, it can be highlighted that the adsorption process was characteristic of chemical adsorption, suggesting the as-synthesized Mg-MOF is a promising candidate for VOC adsorption in the wood-drying industry [58].

In brief, during the entire VOC adsorption/desorption process, both physical adsorption and chemical adsorption (the related effective interactions including π−π stacking interactions, hydrogen bonding, instantaneous dipole forces, dispersion forces and London forces, polar effects and so on) simultaneously played a role. The adsorption behaviour between wood-drying VOCs and Mg-MOFs can act as natural diffusions and π–π stacking chemical interactions, etc. The adsorption process can be described as follows: (i) adsorption in the cabined micropores and single-layer adsorption on the inner face of the mesopores; (ii) multi-layer adsorption reactions, chemical bonding and molecular filling in the mesopores; and (iii) capillary condensation and surface adsorption [59]. The thermal desorption process can be also distinguished into three phases. First, the evaporation of VOCs adsorbed on the surface layer of Mg-MOFs. Second, VOCs molecules were desorbed and released in mesopores and micropores during the multi-layer adsorption. Third, the desorption of the remaining VOCs molecules adsorbed in cabined micropores on the inner walls began to occur.

**Figure 5.**
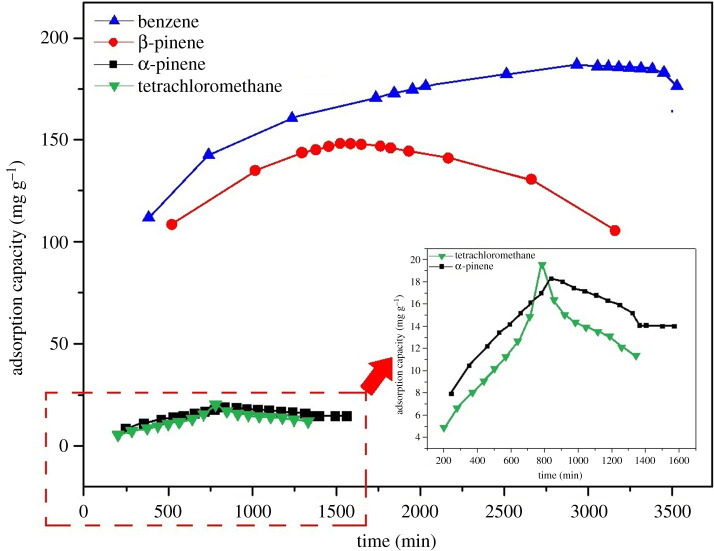
Time-dependent adsorption of vapours of benzene, β-pinene, α-pinene and tetrachloromethane at 298 K on Mg-MOFs.

The effects of different adsorption time on the adsorption capacity of wood-drying VOCs on Mg-MOFs was investigated, which also developed effective π–π stacking interactions between the adsorbates and Mg-MOFs, as presented in figure 5. During the experiments, Mg-MOFs were exposed to a VOC-saturated atmosphere. The adsorption amount was monitored gravimetrically, and the mass of the sample was recorded until the adsorption equilibrium was reached. From figure 5, it can be clearly derived that the trends of the adsorption capacity towards all the VOCs gases increased rapidly in the pre-adsorption stages, but the adsorption efficiency showed a downward trend with increasing adsorption times when the maximum uptakes were reached. The maximum adsorption capacities of the Mg-MOFs were observed at 2932 min, 1513 min, 839 min and 784 min for benzene, β-pinene, α-pinene and tetrachloromethane, respectively. These may be considered a result of the VOCs being introduced to the Mg-MOF surface, followed by spreading into the hierarchical nanopores, thus allowing them to freely and rapidly diffuse into the internal activated sites, and eventually forming a complex with chemical bonding mostly owing to the π–π electrons interactions between Mg-MOFs and VOCs, as compared to the different molecular structures of tetrachloromethane with benzene, β-pinene and α-pinene. Meanwhile, the equilibrium adsorption capacity of four VOCs increases in the order: benzene > β-pinene > tetrachloromethane > α-pinene, which results from the different strengths of chemical bonding interactions between the adsorbate and the surface of Mg-MOFs. Besides, Mg-MOFs performed remarkably well for the examined VOCs, particularly for the capture of benzene and β-pinene compared with reported values in the solid phase adsorbents (electronic supplementary material, table S3). Consequently, this novel as-prepared Mg-MOF has excellent adsorption performance and could be useful in treating VOCs in the wood-drying industry.

## 4. Conclusion

In summary, novel three-dimensional Mg-MOFs with tailored nanostructures were successfully prepared using the solvothermal method. The X-ray crystallography characterization results exhibited a three-dimensional network topology of Mg-MOFs comprising rhombic channels with [Mg_2_(COO)_2_] as the vertex and Bpdc as the edges with diagonal distances of 15.733 Å × 23.736 Å. The results from the N_2_ adsorption/desorption isotherms demonstrated that Mg-MOFs have a high surface area and abundant micro- and mesopores. Benzene, β-pinene, α-pinene and tetrachloromethane were selected as typical VOCs to evaluate the potential application of Mg-MOFs as a VOCs adsorbent. The adsorption capacity for benzene (182.26 mg g^−1^) and β-pinene (144.42 mg g^−1^) is larger than that of α-pinene (18.32 mg g^−1^) and tetrachloromethane (19.60 mg g^−1^) owing to synergetic effects, including natural diffusion, pore-filling and steric hindrance, and were accompanied by chemical adsorption. In this work, the facile and versatile synthesis method and mechanism explored are highly important to the large-scale fabrication of various porous MOFs, which is advantageous for their applications in adsorption and separation involving VOC guest molecules.

## Data Availability

The following are available online at http://www.ccdc.cam.ac.uk, table 1: crystallographic data for Mg-MOFs, table 2: selected bond lengths (Å) and bond angles (°) for Mg-MOFs. See https://doi.org/10.5061/dryad.95x69p8md [60].
